# The association between HPV infection and endometriosis: Risk and fertility outcomes

**DOI:** 10.7555/JBR.39.20250194

**Published:** 2026-07-28

**Authors:** Wenqu Li, Zhenlong Wang, Huizhi Qi, Chengjie Xia, Feixue Chen, Juan Xu, Lei Zhang, Xuemei Jia

**Affiliations:** 1 Department of Gynecology, Women's Hospital of Nanjing Medical University, Nanjing Women and Children's Healthcare Hospital, Nanjing, Jiangsu 210004, China; 2 Nanjing Medical Key Laboratory of Female Fertility Preservation and Restoration, Nanjing, Jiangsu 210004, China

**Keywords:** human papillomavirus, endometriosis, female infertility, pregnancy outcomes, meta-analysis, case-control analysis

## Abstract

While infections have been implicated in endometriosis pathogenesis, the role of human papillomavirus (HPV) remains unclear. This study combined a meta-analysis of seven studies with a case-control study (*n* = 432 surgically treated patients) to evaluate the association of HPV infection with endometriosis risk and fertility outcomes. The meta-analysis showed no significant association between HPV infection and endometriosis risk, either for any HPV subtype (pooled odds ratio [OR] = 2.60, 95% confidence interval [CI]: 0.28–23.87) or high-risk subtypes (OR = 1.68, 95% CI: 0.49–5.75). Notably, HPV prevalence was higher in patients with endometriosis (46% overall; 36% for high-risk subtypes) than in the general population. Evidence regarding the association between HPV infection and fertility outcomes remains limited, with only two eligible studies identified. Our case-control analysis revealed that HPV-positive patients had significantly reduced postoperative live birth rates compared with HPV-negative counterparts (10.6% *vs.* 21.0%, *P* < 0.05). These findings suggest that HPV infection is unlikely to be a major risk factor for endometriosis development, but it may be associated with poorer postsurgical fertility outcomes. The dual-method approach strengthens the evidence for HPV's clinical impact on reproductive prognosis in patients with existing endometriosis.

## Introduction

Declining fertility is one of the key health issues affecting birth rates. It is crucial to investigate the risk of gynecological disorders that affect female fertility to safeguard women's reproductive health, promote reproductive health throughout life, and ensure population stability.

Endometriosis is a benign gynecological disorder characterized by the growth of endometrial tissue outside the uterus. It affects 10%–15% of women of reproductive age and is one of the major contributors to female infertility. A study from 2008 reported that approximately 70.6% of women with endometriosis experienced fertility problems, with an infertility rate of 18.9%^[1]^. Another controlled study revealed a sixfold increase in the prevalence of infertility or subfertility among endometriosis patients compared with controls^[2]^. Women with endometriosis may exhibit abnormal oocyte development and impaired endometrial receptivity, making them more susceptible to infertility, fatigue, multisite pain, and other complications^[3]^. Despite these findings, the risk factors influencing fertility in women with endometriosis remain unclear.

Increasing evidence suggests a close association between infections and the onset and progression of endometriosis, as well as its associated infertility^[4]^. For instance, *Fusobacterium* infection may promote the development of ovarian endometriosis^[5]^, and eradicating this bacterium could be a potential treatment for the condition. Additionally, human papillomavirus (HPV) infections originating in the cervix can spread to the endometrium because of anatomical proximity, thereby increasing the risk of endometrial HPV infection. Furthermore, HPV infections are recognized for impairing male fertility by decreasing sperm motility and increasing sperm DNA fragmentation^[6–7]^. However, clinical evidence is insufficient to confirm whether HPV infection affects fertility in female patients.

Meanwhile, evidence has indicated a correlation between endometriosis and HPV infection, with the prevalence of endometriosis significantly higher among HPV-positive individuals compared with HPV-negative individuals^[8]^. Additionally, studies have demonstrated that infertile women exhibit a greater likelihood of HPV-related cervical cytological abnormalities^[9]^. Furthermore, female HPV infection reduces the success rate of *in vitro* fertilization^[10]^, increases the risk of miscarriage, and leads to other adverse pregnancy outcomes, such as lower live birth rates^[8,11]^.

Nevertheless, other studies have found no significant difference in the probability of HPV infection between women who conceived through *in vitro* fertilization due to tubal or partner factors and those who did not become pregnant^[12]^. While the HPV genotype may influence fertility outcomes, limited data and small sample sizes have impeded definitive conclusions. Epidemiological evidence suggests associations between infectious agents and endometriosis development, between infectious agents and endometriosis-related infertility, and between HPV infection and endometriosis; however, the specific impact of HPV infection on pregnancy outcomes in affected patients remains unclear. Therefore, further investigation is warranted to determine whether HPV infection is associated with adverse fertility outcomes in women with endometriosis. This study aimed to investigate the correlation between HPV infection and the incidence of endometriosis and infertility, providing important evidence for clinical interventions in managing endometriosis-related infertility.

## Materials and methods

### Meta-analysis

#### Search strategy

This review was registered with PROSPERO (CRD420250651884). We systematically searched PubMed, EMBASE, and Web of Science for articles published from their inception to September 2024, with no language or publication status restrictions. The search strategy was developed using a combination of Medical Subject Headings (MeSH) terms and free-text keywords, adapted for each database. For the concept of endometriosis, we used "Endometriosis"[MeSH] and "Endometrioma"[MeSH], supplemented by title/abstract searches for "endometriosis" and "endometrioma". For the viral component, we applied "Papillomaviridae"[MeSH] along with the free-text terms "human papillomavirus" and "HPV". The Boolean operator AND was used to combine the two thematic groups. Additionally, we manually screened the reference lists of all included studies and relevant reviews using the snowballing method to identify any additional eligible records not captured by the electronic databases. The full search algorithms, including all field tags and database-specific syntax, are provided in ***Supplementary Table 1***.

#### Study selection and data collection

We considered case-control, cross-sectional, and cohort studies eligible for inclusion. The study population included women aged 20–55 years, with no restrictions on race, geographic region, or sample size. In cases of duplicate publications, the version with the most comprehensive data was retained. Endometriosis research was accepted, irrespective of the presence of a control group. All types and locations of endometriosis, along with their associated symptoms, met the inclusion criteria in the initial search. The diagnosis of endometriosis was confirmed through laparoscopy or self-reported questionnaires, whereas HPV infection was identified using HPV DNA testing of reproductive tract samples, without any restrictions on HPV genotypes. HPV types are classified as high-risk and low-risk according to their carcinogenic potential. High-risk HPV strains include 16, 18, 26, 31, 33, 35, 39, 45, 51, 52, 53, 56, 58, 59, 66, 68, 73, and 82. Low-risk HPV strains include 3, 6, 11, 30, 34, 35, 40, 42, 43, 44, 54, 55, 61, 62, 64, 67, 69, 70, 71, 72, 74, 81, 83, 84, 89, 90, 91, and IS39. Patients infected with both low-risk and high-risk HPV were classified as having a high-risk HPV infection. Primary outcomes evaluated the prevalence of HPV infection in women with endometriosis or its association with the disease. Additionally, to investigate the association between HPV infection and pregnancy outcomes in endometriosis patients, studies reporting relevant pregnancy data were included in the initial search.

Studies were excluded if they were case series, case reports, of low methodological quality (as evaluated by predefined criteria), or lacked sufficient data. Two investigators independently reviewed the titles and abstracts, excluding irrelevant content. Any discrepancies were resolved through discussion among the authors.

#### Data item and synthesis

Data were extracted and recorded using a standardized form that included general information (first author, publication year, title, investigation period and region, and study phase), study characteristics (number of participants, study design, and sampling methods), participant characteristics (age, sexual history, diagnostic methods for endometriosis, HPV diagnostic methods and typing, history of HPV vaccination, and presence of infertility), and study outcomes (number of HPV infections in the case group [endometriosis group] and control group [non-endometriosis group] and number of infertility cases in the endometriosis group). The primary outcome was the influence of HPV infection on the development of endometriosis and pregnancy in patients with endometriosis, while the secondary outcome was the HPV infection rate in the case groups.

The statistical measure used to estimate the overall prevalence of HPV infection among individuals with endometriosis was the infection rate within the case group. To evaluate the association between HPV infection and the risk of endometriosis and infertility, odds ratios (ORs) were calculated to determine the differences in HPV infection rates between the case and control groups, as well as between the infertility group and the normal pregnancy group within the endometriosis population.

The meta-analysis was conducted using R software (version 4.4.2) and the Meta package. Statistical effect measures were logarithmically transformed, and the inverse-variance method was applied. The pooled HPV prevalence in endometriosis was estimated using a random-effects Poisson-log-normal model. Heterogeneity among studies was assessed using Cochrane's Q test in conjunction with the I^2^ statistic. A fixed-effects model was selected if *P* > 0.1 and I^2^ < 50%. If *P* ≤ 0.1 and I^2^ > 50%, substantial heterogeneity was considered present, and its sources were explored. Due to the limited number of included studies, subgroup analysis and publication bias assessments were not conducted. The significance level was set at *α* = 0.05.

#### Quality assessment and risk of bias

In the current study, the methodological quality of the included studies was rigorously assessed, and the items outlined in the PRISMA statement were strictly followed. Due to the inclusion of fewer than 10 studies^[13–19]^, publication bias could not be evaluated using Egger's test, which limited the statistical power of the analysis. The quality assessment of the included studies was independently conducted by two investigators, with cross-verification. In cases of disagreement, a third investigator was consulted to resolve the discrepancies.

For cross-sectional studies^[16–19]^, research quality was assessed using the quality assessment scale recommended by the Agency for Healthcare Research and Quality (AHRQ). This scale comprises 11 items, each of which is answered with "yes", "no", or "unclear". The scores range from zero to 11, with 8–11 stars indicating high quality, 4–7 indicating moderate quality, and 0–3 indicating low quality.

For case-control studies^[13–15]^, study quality was assessed using the Newcastle-Ottawa Scale (NOS), which evaluates three domains: the selection of cases and controls, their comparability, and the assessment of exposure. Scores range from zero to 9, with 7–9 stars indicating high quality, 4–6 indicating moderate quality, and 0–3 indicating low quality.

### Case-control study

#### Study subjects and grouping

A case-control study was conducted to analyze the clinical data of patients who underwent surgical treatment for endometriosis at Nanjing Maternity and Child Health Care Hospital (Women's Hospital of Nanjing Medical University) between January 2017 and June 2022. Patients were categorized into two groups based on their HPV infection status: an HPV-negative group and an HPV-positive group, the latter further subdivided into high-risk and low-risk HPV-positive subgroups.

#### Inclusion and exclusion criteria

The inclusion criteria were: (1) women of reproductive age ≤ 40 years at the time of surgery; and (2) a histopathologically confirmed diagnosis of endometriosis. The exclusion criteria were: (1) coexisting conditions significantly affecting fertility (*e.g.*, adenomyosis, uterine fibroids); (2) concurrent malignancies (*e.g.*, endometrial cancer, cervical cancer, or ovarian cancer); (3) loss to follow-up post-surgery due to personal reasons; and (4) incomplete clinical data.

#### Research methods

HPV infection was detected by HPV DNA testing, the WHO-recommended primary test. The patient was positioned in the lithotomy position. The cervix was exposed using a speculum, and cervical secretions were collected using an HPV sampling brush. The HPV nucleic acid detection and genotyping kit (PCR capillary electrophoresis fragment analysis) (Health Genetech Co., Ltd., Guangzhou, China) was used to detect HPV DNA in cervical secretions, including high-risk HPV types 16, 18, 26, 31, 33, 35, 39, 45, 51, 52, 53, 56, 58, 59, 66, 68, 73, and 82, as well as low-risk HPV types 6, 11, 42, 43, 44, 81, and 83, according to the manufacturer's instructions. Patients were considered positive for high-risk HPV if infected with at least one high-risk HPV type.

The intraoperative scoring and staging of endometriosis were performed based on the revised American Fertility Society (rAFS) classification, wherein points were allocated as follows: for endometriotic lesions, superficial peritoneal implants < 1 cm, 1–3 cm, and > 3 cm received 1, 2, and 4 points, respectively, while deep peritoneal implants of corresponding sizes received 2, 4, and 6 points; superficial ovarian implants < 1 cm, 1–3 cm, and > 3 cm scored 1, 2, and 4 points per side, respectively, and deep ovarian implants received 4, 16, and 20 points; posterior cul-de-sac obliteration contributed 4 points for partial and 40 points for complete closure; adhesions were scored based on density and extent, with thin adhesions scoring 1, 2, or 4 points and dense adhesions scoring 4, 8, or 16 points for < 1/3, 1/3–2/3, or > 2/3 enclosure of each adnexal structure (ovaries and fallopian tubes), respectively, and complete enclosure of the fimbriated end automatically scored 16 points; the total score was categorized into stage Ⅰ (1–5), stage Ⅱ (6–15), stage Ⅲ (16–40), or stage Ⅳ (> 40)^[20]^.

The endometriosis fertility index (EFI) was calculated according to the criteria summarized and refined by Adamson *et al*^[21]^, which comprise two components: the total history factor score and the total surgical factor score. The total surgical factor score includes both the lowest functioning (LF) score and the r-AFS score. The left and right fallopian tubes and ovaries are scored individually, and the LF score is determined by summing the lowest scores from each side. If one ovary is absent, the LF score is calculated as twice the lowest score of the contralateral side.

Preoperative cancer antigen 125 (CA125) concentrations were measured using the chemiluminescent reagent kit (Cat. #07026986190, Roche, Basel, Switzerland) by a Cobas 6000 analyzer (Roche). Briefly, after a first 12 µL-sample incubation with biotinylated and ruthenium-labeled CA125 antibodies to form the sandwich complex, streptavidin-coated microparticles were added in a second step to bind the complex *via* biotin-streptavidin. The microparticles were then magnetically immobilized on the electrode surface. After washing, the voltage-stimulated chemiluminescence was measured. Results were calculated from an instrument-specific two-point calibration curve and the cobas-link master curve.

#### Statistical methods

Data processing and statistical analysis were performed using SPSS software (version 26.0). Continuous variables that were not normally distributed were presented as medians with interquartile ranges (25th–75th percentiles), and intergroup comparisons were performed using the Mann-Whitney *U* test. Categorical variables were expressed as frequencies and percentages, and group comparisons were assessed using the chi-square test. For 2 × 2 contingency tables, Pearson's chi-square test was applied when the minimum expected count was ≥ 5 and the total sample size (*n*) exceeded 40; otherwise, Fisher's exact test was used. A two-sided *P* < 0.05 was considered statistically significant.

## Results

### Meta-analysis of HPV infection and endometriosis

**Figure 1:**
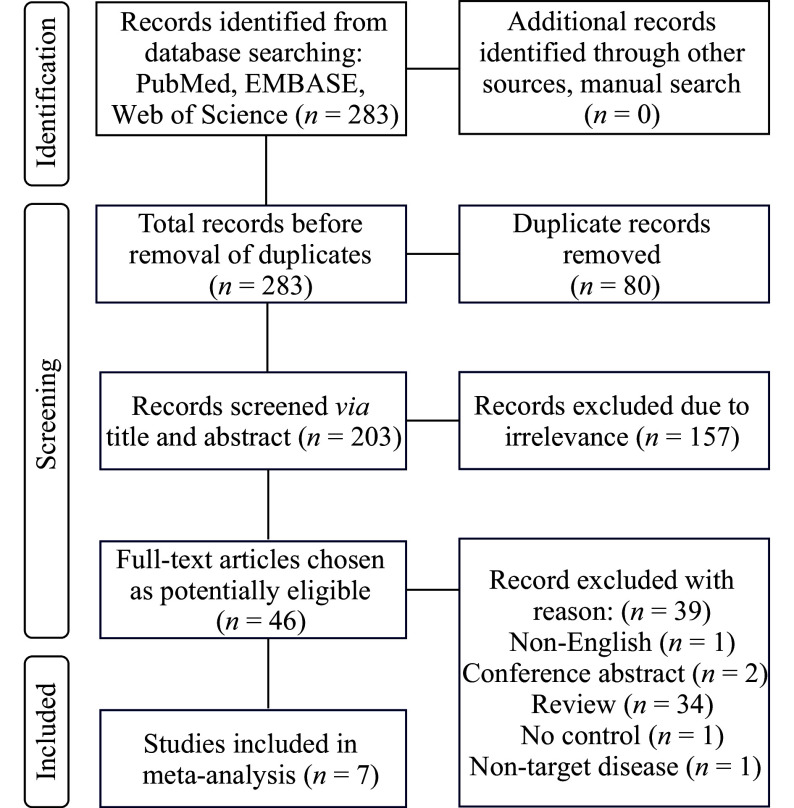
Flow diagram of studies identified, included, and excluded.

To investigate the association between HPV infection and endometriosis risk and infertility, we initially searched for published articles on HPV infection, endometriosis, and fertility outcomes in patients with endometriosis, yielding a total of 283 articles. After removing 80 duplicates, 203 studies were screened based on their titles, abstracts, and relevance, and 46 were identified as potentially eligible for inclusion and were retrieved in full text. Among these, 39 studies were excluded for not meeting the inclusion criteria, including one without a control group^[22]^ and another with an irrelevant target disease^[8]^. Seven studies met the qualitative and quantitative criteria for our meta-analysis^[13–19]^ (***Fig. 1***). However, only two articles explored the association between overall HPV infection and infertility in patients with endometriosis. We first conducted a meta-analysis to assess the association between HPV infection and endometriosis risk, between high-risk HPV infection and endometriosis risk, and between HPV infection and infertility in patients with endometriosis. The main characteristics of the included studies are described in ***Table 1***.

**Table 1:** Characteristics of included studies

Study	Hong *et al* (2023)^[17]^	Moslehi *et al* (2023)^[18]^	Oppelt *et al* (2010)^[13]^	Heidarpour *et al* (2017)^[16]^	Rocha *et al* (2019)^[15]^	Okyay *et al* (2023)^[19]^	Vestergaard *et al* (2010)^[14]^
Country	United States	Iran	Germany	Iran	Brazil	Turkey	Denmark
Design	Cross-sectional	Cross-sectional	Case-control	Cross-sectional	Case-control	Cross-sectional	Case-control
With endometriosis	129	81	56	50	29	410	32
HPV-positive	57	20	14	13	24	202	1
HPV-negative	72	61	15	37	5	208	31
Not available	0	0	27	0	0	0	0
Without endometriosis	1639	–	13	49	31	–	20
HPV-positive	765	–	9	5	11	–	2
HPV-negative	874	–	0	44	20	–	18
Not available	0	–	4	0	0	–	0
High-risk HPV types	16, 18, 26, 31, 33, 35, 39, 45, 51, 52, 53, 56, 58, 59, 66, 68, 73, 82	16, 18, 35, 51, 52, 53, 68	16, 18, 31, 33, 35, 39, 45, 51, 52, 56, 58, 59, 68	16, 18, 31, 33, 35, 39, 45, 52, 56, 58, 59	16, 18, 31, 33, 35, 39, 45, 51, 52, 53, 56, 58, 59, 66, 68, 73, 82	16, 18, 31, 33, 35, 39, 45, 51, 52, 56, 58, 59, 66, 68	35, 68
Low-risk HPV types	6, 11, 40, 42, 54, 55, 61, 62, 64, 67, 69, 70, 71, 72, 81, 83, 84, 89, IS39	3,6,11,40	6, 11, 42, 43, 44	–	6, 11, 30, 34, 40, 42, 43, 44, 54, 55, 61, 62, 64, 67, 69, 70, 72, 74, 81, 83, 84, 91	–	70, 90
HPV detection method	Linear Array HPV Genotyping Test	HPV Direct Flow CHIP	PCR-based ELISA	HPV High-risk Typing PCR Kit	Single-target PCR	Cobas 4800 HPV Test	PCR using the degenerate FAP primer pair
Diagnosis methods	Questionnaire	Clinical symptoms, surgery, or imaging	Surgery	Surgery	Surgery	Surgery or imaging	Surgery
Quality	Moderate	Moderate	Moderate	Moderate	Moderate	Moderate	Moderate
En dash (–) indicates data not available.Abbreviations: ELISA, enzyme-linked immunosorbent assay; FAP, fibroblast activation protein; HPV, human papillomavirus.							

#### No significant difference in HPV positivity between endometriosis patients and controls

**Figure 2:**
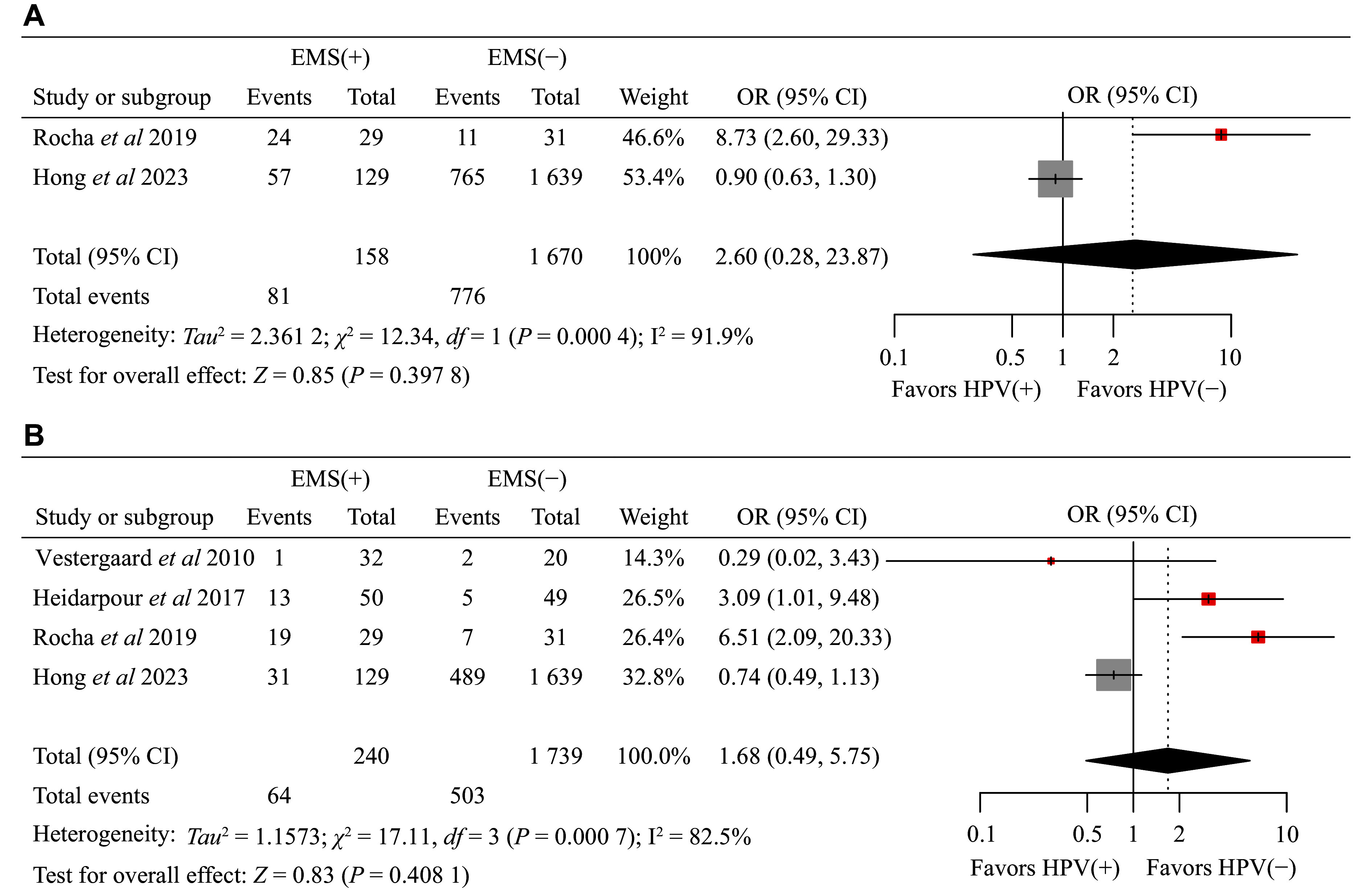
The associations of overall human papillomavirus (HPV) infection (A) and high-risk HPV infection (B) with endometriosis (EMS). Odds ratios (ORs) with 95% confidence intervals (CIs) were calculated using inverse-variance weighting. The area of each square corresponds to the study-specific weight in the meta-analysis. Horizontal lines span the 95% CI range. Solid vertical ticks indicate point estimates. The red squares indicate studies with smaller sample size (*n* < 100), whose wider confidence intervals indicate lower precision. The diamond reflects the pooled estimate with heterogeneity. Studies are labeled by the first author and publication year.

Current evidence on overall rates of HPV infection in individuals with endometriosis remains limited. Our systematic review identified only two comparative studies evaluating the overall HPV infection rates and four studies specifically evaluating the high-risk HPV infection rates in control and endometriosis patients. Using random-effects meta-analysis models, we found no statistically significant difference in either overall HPV infection rates (OR: 2.60, 95% confidence interval [CI]: 0.28–23.87) or high-risk HPV infection rates (OR: 1.68, 95% CI: 0.49–5.75) with respect to endometriosis risk (***Fig. 2***). These non-significant associations indicate that current evidence does not sufficiently support an epidemiological association between HPV infection and endometriosis risk.

**Figure 3:**
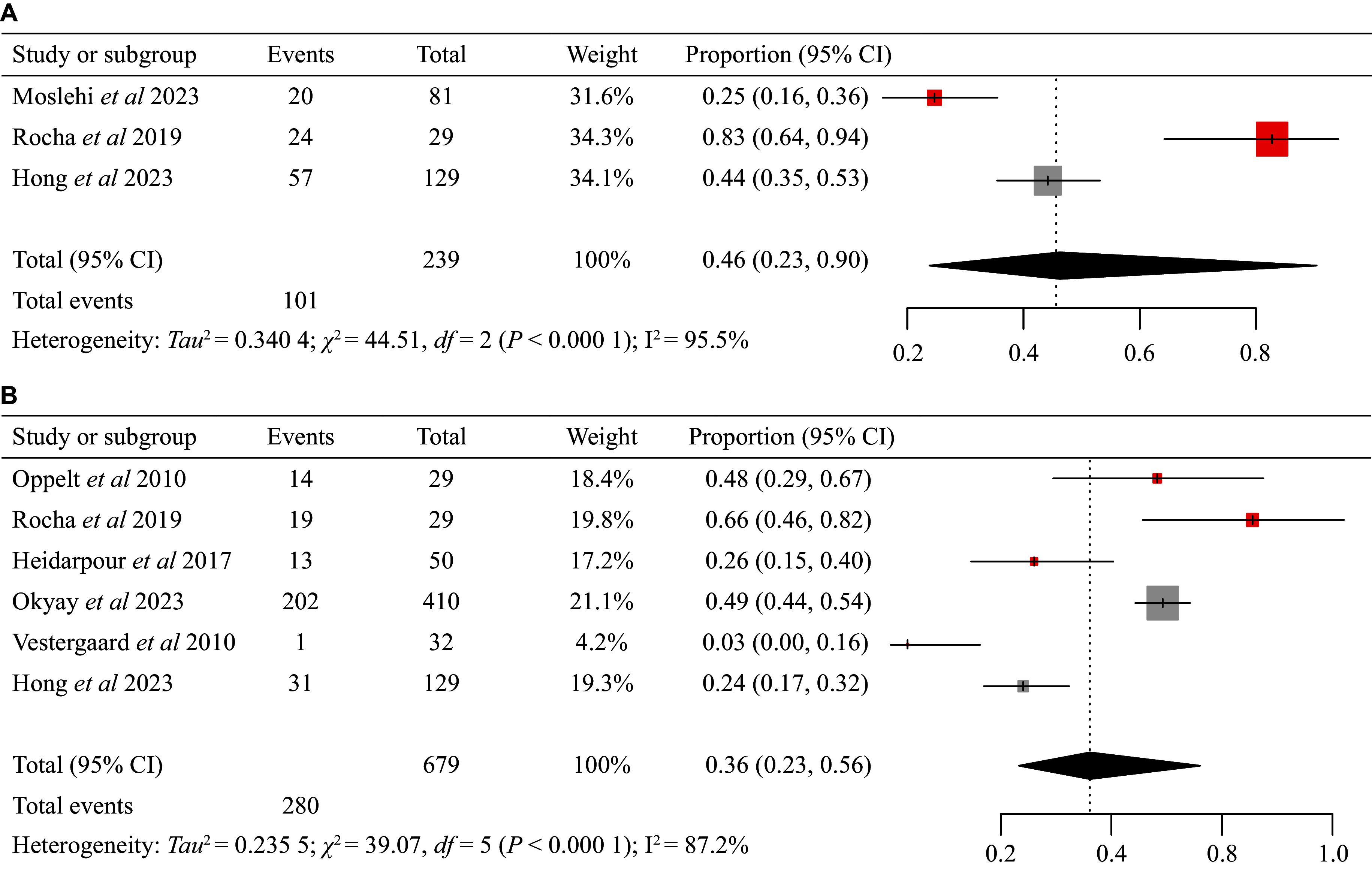
Forest plot analysis comparing prevalence rates of overall human papillomavirus (HPV) infection (A) and high-risk HPV infection (B) among women with endometriosis. Proportions with 95% confidence intervals (CIs) were calculated using a random-effects Poisson-log-normal model. The area of each square corresponds to the study-specific weight in the meta-analysis. Horizontal lines span the 95% CI range. Solid vertical ticks indicate point estimates. The red squares denote studies with smaller samples (*n* < 100), whose wider confidence intervals indicate lower precision. The diamond reflects the pooled estimate with heterogeneity. Studies are labeled by the first author and publication year.

Regarding absolute prevalence, our analysis incorporated three studies comprising 239 endometriosis patients, among whom 101 cases (42.3%) were HPV-positive, and six studies comprising 679 endometriosis patients, among whom 280 cases (41.2%) were infected with high-risk HPV. The random-effects model estimated a pooled HPV infection prevalence of 46% (95% CI: 0.23–0.90) and a pooled high-risk HPV infection prevalence of 36% (95% CI: 0.23–0.56) in endometriosis patients (***Fig. 3***).

#### Conflicting evidence on HPV infection and endometriosis-associated infertility

**Figure 4:**
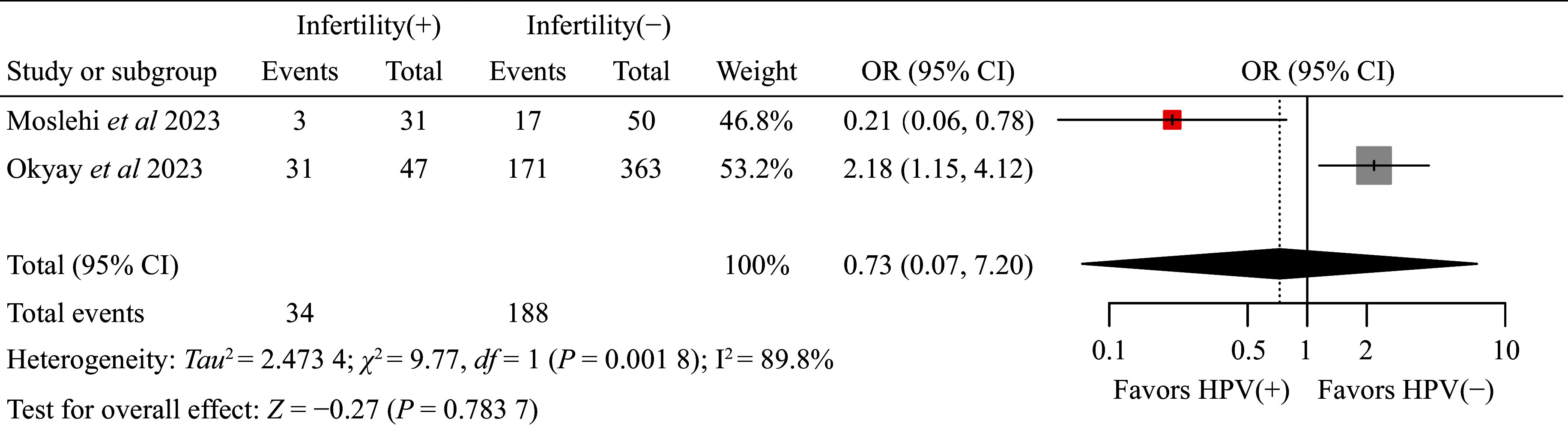
Human papillomavirus (HPV) infection and infertility risk in endometriosis: a random-effects meta-analysis of two cross-sectional studies. Odds ratios (ORs) with 95% confidence intervals (CIs) were calculated using inverse-variance weighting. The area of each square corresponds to the study-specific weight in the meta-analysis. Horizontal lines span the 95% CI range. Solid vertical ticks indicate point estimates. The red squares denote studies with smaller samples (*n* < 100), whose wider confidence intervals indicate lower precision. The diamond reflects the pooled estimate with heterogeneity. Studies are labeled by the first author and publication year.

Current research presents contradictory findings regarding the association between HPV infection and infertility in endometriosis patients^[18–19]^. Our analysis identified two studies with opposing conclusions. A random-effects meta-analysis revealed no statistically significant difference in HPV infection rates between the infertility group of patients with endometriosis and those with normal pregnancies (OR: 0.73, 95% CI: 0.07–7.20) (***Fig. 4***). Considering the small sample sizes in both studies, further investigation into the association between HPV infection and infertility in patients with endometriosis is warranted.

### Case-control analysis of HPV infection and live birth rate

#### Lower postoperative live birth rate in HPV-positive patients

To further investigate the effects of HPV infection on the fertility of women with endometriosis, we conducted a case-control study. A total of 432 patients with endometriosis were included, 66 of whom were infected with HPV before surgery. This group comprised 49 patients with high-risk HPV infections (including 10 patients with HPV types 16 and 18, four of whom had mixed infections), 13 patients with low-risk HPV infections, and four patients with both high-risk and low-risk HPV infections. The remaining 366 patients were in the HPV-negative group. Baseline characteristics, including age, endometriosis score, EFI, CA125 levels, unilateral and bilateral disease, and endometriosis stage, were compared between the two groups. No statistically significant differences were found (*P* > 0.05 for all) (***Table 2***).

**Table 2:** Association of HPV coinfection with clinicopathological features and fertility outcomes in endometriosis patients

Variables	HPV-positive group (*n* = 66)	HPV-negative group (*n* = 366)	*P* value
Age [years, median (Q1, Q3)]	32 (30.0, 36.0)	31 (28.0, 36.0)	0.360^a^
Endometriosis scoring [points, median (Q1, Q3)]	40 (32.0, 73.0)	40 (28.0, 73.0)	0.526^a^
EFI [points, median (Q1, Q3)]	8.0 (7.0, 9.0)	8.0 (7.0, 9.0)	0.569^a^
CA125 [U/mL, median (Q1, Q3)]	50.7 (32.8, 77.6)	46.7 (29.9, 77.2)	0.795^a^
Affected area [*n* (%)]			
One	44 (66.7)	241 (65.8)	0.897^b^
Both	22 (33.3)	125 (34.2)	
Endometriosis staging [*n* (%)]			
Stage 3	36 (54.5)	181 (49.5)	0.446^b^
Stage 4	30 (45.5)	185 (50.5)	
Infertility clinic visit [*n* (%)]			
Yes	12 (18.2)	52 (14.2)	0.403^b^
No	54 (81.8)	314 (85.8)	
Postoperative pregnancy [*n* (%)]			
Yes	10 (15.2)	89 (24.3)	0.103^b^
No	56 (84.8)	277 (75.7)	
Postoperative birth [*n* (%)]			
Yes	7 (10.6)	77 (21.0)	**0.049** ^b^
No	59 (89.4)	289 (79.0)	
Data are presented as median (Q1, Q3) or *n* (%). Statistical methods: ^a^Mann-Whitney *U* test, ^b^chi-square test. Bold indicates statistical significance.Abbreviations: CA125, cancer antigen 125; EFI, endometriosis fertility index; HPV, human papillomavirus.			

However, the postoperative pregnancy rate was lower in the HPV-positive group compared with the HPV-negative group (15.2% *vs.* 24.3%), and the infertility clinic visit rate was higher in the HPV-positive group than in the HPV-negative group (18.2% *vs.* 14.2%), although these differences did not reach statistical significance (*P* = 0.103 and *P* = 0.403, respectively). In contrast, the postoperative live birth rate was significantly lower in the HPV-positive group than in the HPV-negative group (10.6% *vs*. 21.0%; *P* = 0.049) (***Table 2***).

#### No significant difference in live birth rate by HPV type (low-risk *vs.* high-risk)

Subgroup analysis of endometriosis patients with low-risk (13 patients) and high-risk HPV infections (53 patients) revealed that the postoperative live birth rate was higher in the low-risk group (15.4% *vs.* 9.4%). However, this difference was not statistically significant (*P* > 0.05) (***Table 3***).

**Table 3:** Differential effects of high-risk and low-risk HPV infection on clinicopathology and fertility in women with endometriosis and concurrent HPV infection

Variables	Low-risk HPV group (*n* = 13)	High-risk HPV group (*n* = 53)	*P* value
Age [years, median (Q1, Q3)]	33 (30.5, 36.5)	32 (29.5, 36.0)	0.571^a^
Endometriosis Scoring [points, median (Q1, Q3)]	36 (28.0, 84.0)	40 (32.0, 70.0)	0.577^a^
EFI [points, median (Q1, Q3)]	8.0 (6.5, 8.0)	8.0 (7.0, 9.0)	0.173^a^
CA125 [U/mL, median (Q1, Q3)]	36.0 (20.8, 70.8)	50.8 (34.1, 82.0)	0.249^a^
Affected area [*n* (%)]			
One	10 (76.9)	34 (64.2)	0.518^b^
Both	3 (23.1)	19 (35.8)	
Endometriosis staging [*n* (%)]			
Stage 3	8 (61.5)	28 (52.8)	0.572^c^
Stage 4	5 (38.5)	25 (47.2)	
Infertility clinic visit [*n* (%)]			
Yes	3 (23.1)	9 (17.0)	0.691^b^
No	10 (76.9)	44 (83.0)	
Postoperative pregnancy [*n* (%)]			
Yes	2 (15.4)	8 (15.1)	1.000^b^
No	11 (84.6)	45 (84.9)	
Postoperative live birth [*n* (%)]			
Yes	2 (15.4)	5 (9.4)	0.617^b^
No	11 (84.6)	48 (90.6)	
Data are presented as median (Q1, Q3) or *n* (%). Statistical methods: ^a^Mann-Whitney *U* test,^ b^Fisher's exact test, ^c^chi-square test. These results should be interpreted cautiously because of the limited sample size.Abbreviations: CA125, cancer antigen 125; EFI, endometriosis fertility index; HPV, human papillomavirus.			

#### High-risk HPV coinfection correlated with a 24.3% reduction in live birth rate

A further analysis of pregnancy outcomes in endometriosis patients with successful postoperative pregnancies revealed that the live birth rate was lower in those with high-risk HPV infection compared with patients with no HPV infection or low-risk HPV infection (62.5% *vs.* 86.8%). However, the difference was not statistically significant (*P* = 0.099) (***Table 4***).

**Table 4:** Effect of high-risk HPV infection on postsurgical live birth outcomes in endometriosis patients achieving pregnancy

Variable	HPV-negative and low-risk HPV infection group (*n* = 91)	High-risk HPV infection group (*n* = 8)	*P* value
Postoperative live birth [*n* (%)]			
Yes	79 (86.8)	5 (62.5)	0.099
No	12 (13.2)	3 (37.5)	
Data are presented as *n* (%). Fisher's exact test was used.Abbreviation: HPV, human papillomavirus.			

## Discussion

It has been suggested that HPV infection may be associated with the development of endometriosis^[8]^. HPV infection has been detected in both the upper and lower genital tracts of infertile patients and those with endometriosis^[15]^. Notably, two studies that detected HPV infection within endometriosis lesions indicated that HPV might ascend through the genital tract to the uterine cavity, potentially contributing to the development of endometriosis^[13–14]^. However, the results of our meta-analysis revealed no significant association between HPV infection (including high-risk HPV) and endometriosis risk. Further analysis of the prevalence of HPV infection among patients with endometriosis, based on seven studies, indicated that the overall prevalence of HPV infection in patients with endometriosis was 46%, with a 36% prevalence of high-risk HPV infection. While the reported overall HPV infection rate in women ranges from 11.5% to 13.1%, with high-risk HPV infection rates varying between 9.67% and 24.1%^[23–27]^, these data suggest that HPV infection may be more prevalent among patients with endometriosis, which is consistent with the findings of Heidarpour *et al*^[16]^, but this analysis is severely underpowered. Nevertheless, significant heterogeneity was observed (I^2^ > 50%, *P* < 0.1), attributable to both clinical and methodological factors. Clinically, across studies, endometriosis patients differed in disease stage, lesion site, and HPV genotype distribution—variables that may reflect distinct local immune milieus and thus affect HPV detectability. Regional variations in baseline HPV prevalence further contributed to the heterogeneity. Methodologically, discrepancies in detection methods and specimen types across studies were also likely sources of effect estimate dispersion. Although a random effects model was applied to mitigate the impact of heterogeneity, the limited number of included studies precluded effective reduction of heterogeneity through sensitivity or subgroup analyses. Collectively, the current available evidence remains insufficient to establish a definitive association between HPV infection and endometriosis. Large-scale studies with standardized protocols and rigorously matched designs are warranted to better define HPV prevalence in endometriosis and clarify its potential etiologic role.

In patients with ovarian-type endometriosis, large ovarian chocolate cysts can compress the ovarian cortex, leading to atrophy and reduced ovarian reserve function^[28]^. The current standard surgical approach for endometriosis involves removing as much of the ectopic tissue as possible, but this may result in adverse outcomes, such as impaired ovarian function, pelvic adhesions, and laparoscopic surgical complications, all of which can affect postoperative pregnancy outcomes. Endometriosis can result in the accumulation of various toxic inflammatory cytokines and active macrophages in the peritoneal fluid and uterus, which induce a chronic inflammatory response in the pelvic cavity^[29]^. This chronic inflammation may contribute to infertility and miscarriage. Additionally, HPV infection may further trigger an immune response or increase the production of proinflammatory cytokines^[30–32]^, potentially contributing to infertility and pregnancy loss.

Nonetheless, limited research exists on the impact of HPV infection on pregnancy rates and fertility in patients with endometriosis. The only two studies on the association between HPV infection and infertility in endometriosis have reached conflicting conclusions^[18–19]^. Key factors underlying these discrepancies include differences in sample size (410 *vs.* 81), HPV sampling sites (cervical scrapings *vs.* exocervical and tissue samples), detection methods (Cobas 4800 HPV Test *vs.* HPV Direct Flow CHIP), and confounding variables. The latter encompass patient age ranges (30–65 years *vs.* 20–50 years), geographic populations (Turkey *vs.* Iran), and HPV genotype coverage (14 high-risk types *vs*. six high-risk and five low-risk types, with HPV types 6 and 11 being the most prevalent in endometriosis patients). Okyay *et al*^[19]^ included only high-risk HPV types in their study and concluded that the infertility rate was significantly higher in the HPV16/18-infected group. In contrast, Moslehi *et al*^[18]^ included 11 HPV types in their study (six of which were high-risk HPV infections), with fewer than 60% of infections involving high-risk HPV types, yet the infertility rate in the HPV-infected group was significantly lower than that in the control group. Considering these discrepancies, we further analyzed the association between HPV infection and infertility in patients with endometriosis in our sample.

Because several studies included in our meta-analysis classified high-risk HPV according to the International Agency for Research on Cancer classification (14 definitive carcinogenic types in group 1 and four possibly carcinogenic types in group 2B), our case-control study adopted the same criteria. Using this definition, we found the following prevalence rates: high-risk HPV in 49/66 (74.2%), low-risk HPV in 13/66 (19.7%), and high-risk/low-risk coinfections in 4/66 (6.1%). The postoperative live birth rate was significantly lower in the HPV-positive group than in the HPV-negative group. Stratified analysis further showed a higher postoperative live birth rate in the low-risk HPV infection group compared with the high-risk HPV infection group. These findings differ from those reported by Moslehi *et al*, but are more closely aligned with the research conducted by Okyay *et al*^[18–19]^, supporting a potential association between high-risk HPV infection and worse fertility outcomes in patients with endometriosis. This observation is also in line with our subgroup analysis, which indicated a lower postoperative live birth rate in the high-risk HPV group compared with the low-risk HPV infection group (9.4% *vs*. 15.4%). Nevertheless, the limited sample size in our study reduced the statistical power of our findings. Further investigations are warranted to elucidate the distinct effects of low-risk versus high-risk HPV infections on the infertility of endometriosis patients.

High-risk HPV infection appears to exert a measurable influence on reproductive outcomes. However, current studies, including our own, are limited by relatively small sample sizes and low statistical power. Furthermore, the results may have been influenced by various confounding factors, including the HPV detection methods, definitions of high-risk HPV, regional variations in HPV genotypes, socioeconomic status (particularly quality of life and marital status), timing of viral exposure, patients' age, and the ongoing fertility intentions of both patients and their family members. These variables likely impact both pregnancy rates and overall fertility outcomes, thereby introducing additional complexity to the interpretation of our findings.

While our meta-analysis demonstrated no etiological association between HPV infection and endometriosis development, the case-control study revealed significantly reduced postoperative live birth rates among HPV-positive endometriosis patients compared with their HPV-negative counterparts. Furthermore, the subgroup analysis showed that the postoperative live-birth rate was higher in the low-risk HPV group than in the high-risk HPV group. These findings indicate that systematically evaluating and managing concurrent HPV infection in patients with endometriosis may help identify women at increased risk of adverse reproductive outcomes, offering new intervention directions for patients with both endometriosis and HPV infection-related infertility. However, the limited sample size in our study reduced the statistical power of these findings. Therefore, future prospective and longitudinal studies with larger sample sizes and comprehensive HPV-genotype analyses are warranted to validate these findings.

## Additional information

The datasets generated or analyzed during the current study are not publicly available as they form part of an ongoing investigation, but are available from the corresponding author upon reasonable request. The online version contains supplementary materials available at http://www.jbr-pub.org.cn/article/doi/10.7555/JBR.39.20250194.
