# Supplementary material for: The association between HPV infection and endometriosis: Risk and fertility outcomes
Source: J Biomed Res. 2026 Jul 28;40(4):383–94. doi: 10.7555/JBR.39.20250194 (PMC13438304; doi:10.7555/JBR.39.20250194)
Supplement: Supplementary file 1 [file jbr-40-4-383-S1.pdf]

Available online at www.jbr-pub.org.cn

Open Access at PubMed Central

JBR

Journal of Biomedical Research, 2026 40(4): 1–2

Supplementary Data

The association between HPV infection and endometriosis: Risk and fertility outcomes

Wenqu Li[1,△], Zhenlong Wang[1,△], Huizhi Qi[1,△], Chengjie Xia[1], Feixue Chen[1], Juan Xu[1,2], Lei Zhang[1,✉], Xuemei Jia[1,2,✉]

[1]*Department of Gynecology, Women's Hospital of Nanjing Medical University, Nanjing Women and Children's Healthcare Hospital, Nanjing, Jiangsu 210004, China;*
[2]*Nanjing Medical Key Laboratory of Female Fertility Preservation and Restoration, Nanjing, Jiangsu 210004, China.*

[△]These authors contributed equally to this work.

[✉]Corresponding authors: Lei Zhang, E-mail: njsfyzl@163.com, ORCID: 0009-0005-9559-7501; Xuemei Jia, E-mail: xmjia@njmu.edu.cn, ORCID: 0000-0002-9323-4425.

Received: 06 May 2025; Revised: 22 September 2025; Accepted: 26 September 2025; Available online: 09 October 2025; Published date: 28 July 2026

CLC number: R711.71, Document code: A

The authors reported no conflicts of interest.

This is an open access article under the Creative Commons Attribution (CC BY 4.0) license, which permits others to distribute, remix, adapt and build upon this work, for commercial use, provided the original work is properly cited.

© 2026 by Journal of Biomedical Research.

https://doi.org/10.7555/JBR.39.20250194

Supplementary Table 1 **Search strategy**

(1) PUBMED

Search	Query
#1 19 200 955	(("Endometriosis"[Mesh]) OR (((Endometrioses) OR (Endometrioma)) OR (Endometrioma))) AND ((((((((((((((Human Papillomavirus Virus) OR (Papillomavirus Virus, Human)) OR (Virus, Human Papillomavirus)) OR (Human Papilloma Virus)) OR (Human Papilloma Viruses)) OR (Papilloma Virus, Human)) OR (Virus, Human Papilloma)) OR (Human Papillomavirus)) OR (Human Papillomaviruses)) OR (HPV Human Papillomavirus)) OR (HPV Human Papillomaviruses)) OR (Human Papillomaviruses, HPV)) OR (Human Papillomavirus, HPV)) OR (HPV, Human Papillomavirus Viruses)) OR ("Human Papillomavirus Viruses"[Mesh]))
#2 20 300 784	(("Endometriosis"[Mesh]) OR (((Endometrioses) OR (Endometrioma)) OR (Endometrioma))) AND ((((((((((((((Human Papillomavirus Virus) OR (Papillomavirus Virus, Human)) OR (Virus, Human Papillomavirus)) OR (Human Papilloma Virus)) OR (Human Papilloma Viruses)) OR (Papilloma Virus, Human)) OR (Virus, Human Papilloma)) OR (Human Papillomavirus)) OR (Human Papillomaviruses)) OR (HPV Human Papillomavirus)) OR (HPV Human Papillomaviruses)) OR (Human Papillomaviruses, HPV)) OR (Human Papillomavirus, HPV)) OR (HPV, Human Papillomavirus Viruses)) OR ("Human Papillomavirus Viruses"[Mesh]))
#3 27 928 852	(("Endometriosis"[Mesh]) OR (((Endometrioses) OR (Endometrioma)) OR (Endometrioma))) AND ((((((((((((((Human Papillomavirus Virus) OR (Papillomavirus Virus, Human)) OR (Virus, Human Papillomavirus)) OR (Human Papilloma Virus)) OR (Human Papilloma Viruses)) OR (Papilloma Virus, Human)) OR (Virus, Human Papilloma)) OR (Human Papillomavirus)) OR (Human Papillomaviruses)) OR (HPV Human Papillomavirus)) OR (HPV Human Papillomaviruses)) OR (Human Papillomaviruses, HPV)) OR (Human Papillomavirus, HPV)) OR (HPV, Human Papillomavirus Viruses)) OR ("Human Papillomavirus Viruses"[Mesh]))
#4 30 940 433	(("Endometriosis"[Mesh]) OR (((Endometrioses) OR (Endometrioma)) OR (Endometrioma))) AND ((((((((((((((Human Papillomavirus Virus) OR (Papillomavirus Virus, Human)) OR (Virus, Human Papillomavirus)) OR (Human Papilloma Virus)) OR (Human Papilloma Viruses)) OR (Papilloma Virus, Human)) OR (Virus, Human Papilloma)) OR (Human Papillomavirus)) OR (Human Papillomaviruses)) OR (HPV Human Papillomavirus)) OR (HPV Human Papillomaviruses)) OR (Human Papillomaviruses, HPV)) OR (Human Papillomavirus, HPV)) OR (HPV, Human Papillomavirus Viruses)) OR ("Human Papillomavirus Viruses"[Mesh]))
#5 37 082 187	(("Endometriosis"[Mesh]) OR (((Endometrioses) OR (Endometrioma)) OR (Endometrioma))) AND ((((((((((((((Human Papillomavirus Virus) OR (Papillomavirus Virus, Human)) OR (Virus, Human Papillomavirus)) OR (Human Papilloma Virus)) OR (Human Papilloma Viruses)) OR (Papilloma Virus, Human)) OR (Virus, Human Papilloma)) OR (Human Papillomavirus)) OR (Human Papillomaviruses)) OR (HPV Human Papillomavirus)) OR (HPV Human Papillomaviruses)) OR (Human Papillomaviruses, HPV)) OR (Human Papillomavirus, HPV)) OR (HPV, Human Papillomavirus Viruses)) OR ("Human Papillomavirus Viruses"[Mesh]))
#6 37 198 431	(("Endometriosis"[Mesh]) OR (((Endometrioses) OR (Endometrioma)) OR (Endometrioma))) AND ((((((((((((((Human Papillomavirus Virus) OR (Papillomavirus Virus, Human)) OR (Virus, Human Papillomavirus)) OR (Human Papilloma Virus)) OR (Human Papilloma Viruses)) OR (Papilloma Virus, Human)) OR (Virus, Human Papilloma)) OR (Human Papillomavirus)) OR (Human Papillomaviruses)) OR (HPV Human Papillomavirus)) OR (HPV Human Papillomaviruses)) OR (Human Papillomaviruses, HPV)) OR (Human Papillomavirus, HPV)) OR (HPV, Human Papillomavirus Viruses)) OR ("Human Papillomavirus Viruses"[Mesh]))

(1) PUBMED

#1 L2027064807	('endometriosis'/exp OR 'endometriosis' OR 'endometrioses' OR 'endometrioma'/exp OR 'endometrioma' OR 'endometriomas'/exp OR 'endometriomas') AND ('human papillomavirus viruses' OR 'human papillomavirus virus' OR 'papillomavirus virus, human' OR 'virus, human papillomavirus' OR 'human papilloma virus' OR 'human papilloma viruses' OR 'papilloma virus, human' OR 'virus, human papilloma' OR 'human papillomavirus' OR 'human papillomaviruses' OR 'hpv human papillomavirus' OR 'hpv human papillomaviruses' OR 'human papillomaviruses, hpv' OR 'human papillomavirus, hpv' OR 'hpv, human papillomavirus viruses')
